# Qualitative Evaluation of General Practitioner Training Program as Viewed by Graduates from Shiraz, Fasa and Jahrom Medical Universities

**Published:** 2015-07

**Authors:** FATEMEH SHAHIDI, MOHAMMAD MEHDI SAQEB, MITRA AMINI, ABOLGHASEM AVAND, HAMID REZA DOWLATKHAH

**Affiliations:** 1 Shiraz University of Medical Sciences, Shiraz, Iran;; 2 Quality Improvement in Clinical Education Research Center, Shiraz University of Medical Sciences, Shiraz, Iran;; 3 English Language Department, Fasa University of Medical Sciences, Fasa, Iran;; 4 Jahrom University of Medical Sciences, Jahrom, Iran;

**Keywords:** Quality, Evaluation, General practitioners, Medical students

## Abstract

**Introduction:**

The majority of countries have brought the quality of higher education into focus in the past few years. They have tried to improve the quality of their own higher education. The studies show that Iranian Universities are not at an accepted level in terms of quality. They have encountered several problems which have diminished their quality level. This study aimed at assessing the quality of medical education program as viewed by general practitioners graduated from Shiraz, Fasa and Jahrom Medical Universities.

**Methods:**

This is a cross-sectional study. 215 subjects were selected based on a census of all the general practitioners graduated from Shiraz, Fasa and Jahrom Universities during 2011-2013. The questionnaire used for collecting the data was that of the Association of Graduates from American Medical Colleges. The collected data were then analyzed using SPSS 14 through which such descriptive and bivariate statistics as percentage, means, Standard Deviation and ANOVA were used. The level of significance was set to 0.05.

**Results:**

The questionnaire return rate was 97%. As to the graduates' preclinical experiences, five indices were studied which were assessed as "average" in graduates' views. However, with respect to their clinical experiences five indices were equally studied, among which such indices as "Communication skills" and "The quality of medical apprenticeship" were evaluated as "desirable" in view of the graduates from the very three universities. On the contrary, the quality of clinical experiences and technological skills was evaluated as "almost weak"; furthermore, the integration of basic science with required clinical experience was also considered "weak".

**Conclusion:**

It seems essential to set up an annual assessment of general practitioner education program and a review of the medical education program in Iran based on the global medical advancement and international standards.

## Introduction

Universities are the most valuable institutes that a society has for its progress and development. On the one hand, universities protect and convey the cultural heritage and dominant values of the society; on the other hand, they are responsive to social needs for acquisition, dissemination and flourishing of knowledge and technology. We would have different expectations of higher education. Due to the advancements in science and technology, they have gradually changed. Currently, higher education is regarded as a major contributing factor in achieving economic, social and cultural development policy, so universities, as major subsystems of higher education and the centers for training qualified, efficient and competent workforce, have a key and vital role in the development process of a country to meet the real needs of the society in various fields. If the main responsibility of higher education is to train and provide the required specialist workforce of the country, this mission is regarded as one of the qualitative objectives which are directly linked to the quality of higher education system (1). One of the fundamental goals of higher education is improvement of quality worldwide (2). The quality of education and research is a topic of concern to university systems. Considerable efforts have been made to improve the quality of higher education and to achieve the objectives of academic systems in many countries in the last two decades (3).  Accordingly, it can be inferred that promotion and improvement of the quality of education (research and specialized services) is a noble objective of any educational system. Unless the objective is realized, it would lead to dissipation of economic resources, decline of the learners’ self-confidence, and insecurity of their personal and social characters (4). Evidence shows that the rising cost of higher education or appropriation of more resources to higher education lead to lower output results from poor quality of higher education and its activities (5). As a result, the majority of countries have brought the quality of higher education into focus in the past few years. They have tried to promote the quality of higher education.

Studies show that, in terms of quality, Iranian universities are not at a desirable and acceptable level (6). They have encountered several problems which have declined their quality level so that the most distinguished trait of Iranian higher education claimed to be the downtrend of quality indices over the past decade (7).

Educational activities of a country are interpreted as investing in education passed from a generation to the future one (8). According to Medical Education International Federation’s report, over the two past decades, we have witnessed the indiscriminate expansion of medical education (at least in general practice section) all over the world. Yet, due to increased community awareness, public expectations of doctors have exceeded. Therefore, it can be said that medical education in our country is in the same boat as what is stated in Medical Education International Federation’s report. This unpleasant situation has led the authorities to take measures as to the quality of general practitioner education program (9).

Dennet and Harden stated that a doctor is a whole collection of varied capabilities, but many medical schools may train doctors, but the nature of their training is uncertain (10). General Medical Council (GMC) in its document as ‘The Doctors of Tomorrow’ emphasized that medical school had better improve the graduates’ readiness for their first-year job entrance (11). On the other hand, the relationship between the people involved in the health care system is changing. If doctors are expected to function effectively, a major revision of their medical education, as students of medicine and would-be professional doctors, has to be undertaken (12). Therefore, evaluation is an integral part of any clinical training (13). Observation of successful educational systems in the world represents the role of internalization of an efficient and effective system of quality evaluation (14).

The graduates are one of the best sources for the evaluation of the performance of educational systems. They are the former newcomers of the universities who have been trained within the very educational system and received the necessary education. Therefore, the performance of an educational system can be objectively reflected in the graduates (15).

Evaluation is the core of any training program (16) and is regarded as one of the most important aspects of educational activities (17).  Evaluation and revision of the curriculum is like a mirror through which decision makers and those involved in education can form an image of how the general process of learning would be. By using this image, along with meeting the individual and social needs, authorities have to promote the quality of the educational programs (18). All these factors necessitate the development of a suitable and efficient evaluation system in higher education corresponding to its characteristics and conditions in order to be aware of the present situation, and improve and promote the quality of education and research. Hence, whenever the betterment and promotion of quality of education and research are considered, it is accepted that there is no satisfaction with the current condition. In other words, we believe the quality of education is not satisfactory, and we need to change it anyhow. The change can be made in such different ways as evaluation- as a basis for understanding the current situation. Global experience in this field is very high and the usefulness of scientific and participatory evaluation methods has been verified in realization of this goal (7).  This study aimed at assessing the quality of the medical curriculum in view of GPs graduated from Shiraz, Fasa, and Jahrom Medical Universities.

## Methods

This is a cross-sectional study conducted at Shiraz, Fasa and Jahrom Universities during 2011-2013 academic years. The 215 subjects in this study were all general practitioners. Before graduation, they had to fill out the questionnaires at the University Education Office. Approved by the Vice-Chancellors for Education of the three Universities, the mandatory criterion for graduation necessitated that they fill out the questionnaires during the last two semesters. In all, 120 questionnaires were filled out at Shiraz University of Medical sciences, 51 at Jahrom University of Medical sciences, and 44 at Fasa University of Medical sciences. The subjects were selected by conducting a census of all graduates of medicine from the above-mentioned universities between June 2012 and June 2013. The questionnaire used for collecting the data was that of the Association of Graduates from American Medical Colleges. This questionnaire is a nationwide one prepared by the Association of American Medical Colleges in 1978 (19). It is a very important tool for medical colleges to evaluate their educational programs and to make use of medical students' experiences in order to improve the educational system. The validity of the questionnaire was approved by ten experts in medical education. An estimate of the reliability of the test scores was achieved through Cronbach's alpha which was 83%, indicating the acceptability of the questionnaire. It consisted of four parts including demographic information, preclinical experiences, clinical experiences, and an open-ended question on the strength and weakness of medical schools.

Having been approved by the experts in education and language, the questionnaire was first translated into Farsi by experts of Medical Education Development Center of Shiraz Medical University. Then, it was back translated into English by university lecturers of English Department of Shiraz Medical University. Having collected the data, the researchers analyzed it by using Statistical Package for the Social Sciences SPSS 14 through which such descriptive and bivariate statistics as percentage, means, Standard Deviation and ANOVA were used. The level of significance was set to 0.05.

Finally, based on operational definition of research, the appropriateness of the current status of medical education at Shiraz, Fasa and Jahrom Medical Universities was determined with the  following levels: A. Very good: if the score is 4–5; Good: if the score is 3–4; Medium : if the score is 2–3; Poor : if the score is 1–2.

Before distributing the questionnaires among the students, they were reassured that the data would be collected exclusively to be used in the research project, remaining nameless. In addition, the same message was printed on top of each questionnaire. 

## Results

There was a significant correlation among the three Medical Universities with respect to preclinical experiences in terms of identifying the learning objectives of basic sciences lectures for the students, appropriate integration of basic sciences contents into basic sciences lectures, giving relevant examples in basic sciences lectures, and preparing the students to enter the internship course (p<0.001). However, there was no significant correlation between learning objectives and assessment tests of basic sciences contents in the three Medical Universities (p<0.05).

 97% of the questionnaires were distributed among the researchers. The demographic data showed that out of 215 participants in this study, 120 graduates (55.8%) were from Shiraz Medical University; 51 (23.7%) from Jahrom Medical University, and 41 (20.5%) from Fasa Medical University. In addition, 97 participants were male (45%) and118 were female (55%).  Out of them, 79 female graduates (65.8%) and 41 male graduates (34.1%) were from Shiraz Medical University; 18 female graduates (40.9%) and 26 male graduates (59.1%) were from Fasa Medical University; and 30 female graduates (58.8%) and 21 male graduates (41.1%) were from Jahrom Medical University. 

With respect to preclinical experiences, five indices were studied, as presented in Table 1. The total score of the five indices was evaluated as ‘average’ in view of the graduates (two to three scores).

**Table 1:** Preclinical experiences

	**Medical Universities**					
**Indices**	**Shiraz**	**Fasa**	**Jahrom**	**Total**	**F**	**p**
	**Mean±SD**	**Mean±SD**	**Mean±SD**	**Mean±SD**		
**The score was obtained from the mean score if specific objectives of basic science lectures were made clear to the medical students**	2.50±0.99	2.42±0.93	3.14±1.00	2.63±1.01	8.935	0.001
**The score was obtained from the mean score if appropriate integration of basic science contents was made into basic science courses**	2.59±0.98	2.63±0.93	3.10±0.96	2.72±0.98	5.200	0.006
**The score was obtained from the mean score if the achievement tests on basic science contents were proportionate to the educational objectives **	2.53±1.02	2.37±0.79	2.82±1.09	2.56±1.00	2.629	0.075
**The score was obtained from the mean score if relevant examples were given in basic science lectures**	2.30±1.08	2.16±0.90	2.88±0.97	2.41±1.04	7.373	0.002
**The score was obtained from the mean score if basic science lectures prepared the medical students to enter apprenticeship**	2.36±1.01	2.23±0.92	3.04±1.15	2.50±1.07	9.481	0.001

The score obtained from the mean score on how to study basic science courses in order to prepare the students to enter the apprenticeship course is presented in Table 2. The minimum score belonged to the biochemistry course (the mean score was 1.87 for three universities) and the maximum score to the anatomy of the limbs (the mean score was 3.14 for three universities).

**Table 2:** The mean score as to how to study basic science textbooks for preparation in apprenticeship course

**Indices**	**Medical Universities**			
	**Shiraz**	**Fasa**	**Jahrom**	**Total**
	**Mean±SD**	**Mean±SD**	**Mean±SD**	**Mean±SD**
**Biochemistry**	1.80±1.00	1.53±0.74	2.31±0.99	1.87±0.98
**Genetics**	1.86±0.83	1.72±0.73	2.45±1.06	1.97±0.91
**Anatomy of the head and neck **	2.73±1.00	2.47±0.98	3.04±0.75	2.74±0.96
**Anatomy of the limbs**	3.09±0.85	3.16±0.87	3.26±0.63	3.14±0.80
**Anatomy of the trunk**	2.94±0.90	2.98±0.74	3.16±0.75	3.0±0.83
**Immunology**	2.62±0.96	2.58±0.93	2.84±0.89	2.66±0.93
**Introduction to clinical medicine**	2.72±0.98	2.49±0.83	2.84±0.92	2.69±0.94
**Histology**	2.37±0.97	2.60±1.00	2.75±1.02	2.50±0.99
**Microbiology**	2.29±0.93	2.40±1.04	2.94±0.85	2.46±0.07
**Pathology**	2.62±0.95	2.49±0.86	2.73±0.97	2.62±0.93
**Pharmacology**	2.42±0.98	2.49±1.03	2.77±0.97	2.51±0.99
**Physiology**	2.85±0.93	2.77±0.97	3.08±0.88	2.88±0.92
**Psychology**	2.33±0.92	1.90±0.82	2.46±0.93	2.27±0.92
**Pathophysiology**	2.86±0.90	2.79±0.91	2.78±0.92	2.82±0.99

Regarding clinical experiences, five indices were studied, among which such indices as 'Communicative Skills' and 'The quality of medical apprenticeship' were assessed as desirable in view of the graduates (The mean score was 3.18 and 3.07, respectively).  However, such indices as 'The quality of clinical experiences' and 'Technological skills' were evaluated as weak (The mean score was 2.5 and 2.76, respectively). Equally, such indices as 'The integration of basic science into required clinical experiences' were scored as weak in the graduates' opinions (The mean score was 2.62) (Table 3).

**Table 3:** Clinical experiences of the participants

**Indices**	**Medical Universities**					
	**Shiraz**	**Fasa**	**Jahrom**	**Total**	**F**	**p**
	**Mean±SD**	**Mean±SD**	**Mean±SD**	**Mean±SD**		
**Integration of basic science lectures into required clinical experiences**	2.62±1.11	2.20±0.80	2.73±0.80	2.56±1.04	3.315	0.038
**The quality of clinical experiences **	2.48±0.55	2.40±0.48	2.60±0.48	2.50±0.55	2.87	0.057
**The quality of medical apprenticeship**	2.9±0.57	3.10±0.42	3.20±0.42	3.07±0.52	4.007	0.020
**Communication skills**	3.20±0.72	3.20±0.61	3.15±0.63	3.18±0.70	0.109	0.896
**Technological skills**	2.82±0.85	2.66±0.62	2.71±0.61	2.76±0.78	0.890	0.412

The mean score on the quality of clinical experiences gained in various clinical wards is shown in Table 4. In view of the subjects, all of the clinical wards were qualitatively poor (The mean score was between 2 and 3 for the three universities). The highest score belonged to the Internal medicine ward (The mean score was 2.87), but the lowest score was related to the Community medicine (The mean score was 1.89).

There was a significant difference among Shiraz, Jahrom and Fasa Medical Universities in terms of the integration of basic sciences lectures into required clinical experiences, the quality of clinical experiences, and the quality of medical internship (p<0.001). However, there was not such a significant difference between communicative and technological skills among them (p<0.05).

**Table 4:** The mean score of the ranking of clinical experiences quality gained in clinical apprenticeship

**Indices**	**Medical Universities**			
	**Shiraz**	**Fasa**	**Jahrom**	**Total**
	**Mean±SD**	**Mean±SD**	**Mean±SD**	**Mean±SD**
**Internal Medicine**	2.65±0.88	2.64±0.79	2.98±0.81	2.73±0.85
**Gynecology **	2.42±0.89	2.37±0.93	2.16±0.90	2.34±0.90
**Pediatrics**	2.81±0.84	2.88±0.78	3.04±0.83	2.87±0.82
**Psychiatry**	2.60±0.80	3.64±0.85	3.14±0.80	3.74±0.84
**Radiology**	2.46±0.88	2.60±0.79	2.59±1.02	2.52±0.89
**Surgery**	2.26±0.89	2.51±0.94	2.75±0.80	2.43±0.89
**Anesthesiology**	2.64±0.95	2.36±0.93	2.63±0.85	2.58±0.92
**Physical Medicine**	2.52±0.96	2.15±0.79	2.84±0.96	2.52±0.95
**Social Medicine**	1.85±0.91	2.05±1.00	1.90±0.88	1.89±0.90

In view of the subjects, the integration of basic science lectures into the required clinical experiences was scored as poor (the mean score was 2.56 for the three universities). The maximum percentage, 72% for the three universities, for making use of evaluation methods at apprenticeship final exam belonged to knowledge test (both in written and computerized form). However, 66% and 60% of the tests belonged to OSCE and logbook, respectively.

**In graduates' opinion, the points of strength of Shiraz Medical University are as follows:**

1. Perfect and effective education courses
2. Too many university teachers
3. Experienced university teachers
4. Empowering the students as to different fields
5. Diversity of patients and observation of manifestations of diseases

**The points of weakness of Shiraz Medical University are as follows**:

- Lack of relationship between basic sciences and clinical courses
- Weakness in teaching of medical ethics and its rules and regulations
- Integration of the contents of pre-clinical into clinical lectures
- Lack of proper practice in dealing with outpatients
- Nurses' lack of respect for medical students
- Poor outpatient training
- Lack of proper evaluation

**In graduates' opinion, the points of strength of Fasa and Jahrom Medical Universities are as follows:**

1. Interns' taking more responsibility due to lack of residents, leading to increased self-confidence
2. Direct involvement of both the interns and  specialists in treatment
3. Lots of communication between teachers and students
4. Observation of  prevalent diseases
5. Independence  of students

**The points of weakness of Fasa and Jahrom Medical Universities are as follows:**

- Little relationship between preclinical and clinical lectures
- Teachers' inability to motivate students
- The small number of full-time teachers
- Making use of young and less experienced teachers
- Inadequacy of patients and diversity of diseases

## Discussion

**5.1. Preclinical Experiences**

The introduction of learning objectives at the beginning of each course helps the teachers to determine the extent at which students are expected to gain knowledge, attitude and skills at the end of the course (20). Furthermore, it paves the way for both the students and the teaching departments to achieve the objectives. The score obtained from the mean scores regarding the specificity of learning objectives of basic science lectures was assessed as ‘average’ in the three universities. This stresses the necessity of informing the students of educational objectives at the beginning of each semester.

The study of KhajehAzad and his colleagues carried out at Baqiyat-Allah Medical University also shows that both learning objectives and job description in Medical Education Curriculum was assessed as ‘very poor’ (21).  However, in the report issued by the Association of American Medical Colleges in 2012, identifying the learning objectives for the students was evaluated to be ‘very favorable’ (22).

In spite of the fact that the medical students are presented with too intensive and extensive lectures in preclinical course, melding of the contents of basic science courses has not been properly made.

In a comprehensive evaluation report on medical education curriculum by Educational Development Center of Tehran University published in 2008, it was claimed that the educational contents of most courses do have a specialized nature, but they do not correspond with the responsibilities a general practitioner is expected to fulfill. Non-compliance of the volume and contents of the textbooks with their values as credits along with their voluminous contents has put a lot of pressure on students (23). In contrast, the report of the Association of American Medical Colleges published in 2011 asserts that the contents of basic sciences have properly been incorporated into basic science courses (22).

Such courses as anatomy of the limbs and the trunk had suitably prepared the students to enter the apprenticeship course; on the contrary, biochemistry and genetics were assessed to be ‘poor’ in preparing the students for apprenticeship course. In the study by Simon Watmoug et al. (2009) on the effectiveness of traditional medical education curriculum in England, it was reported that graduates complained of studying irrelevant subjects. To the graduates, the courses in the very first two years were so boring, especially biochemistry (24). The Association of American Medical Colleges (2012) reported that biochemistry course attained the minimum score (The mean score was 2.6) whereas pathology and anatomy courses obtained the maximum score (The mean score was 3.5 and 4.5, respectively) (22). Since the basic science courses could not properly prepare the students to enter the apprenticeship course, as documented by the report of Educational Development Center of Tehran University (23), it seems that the curriculum of medical basic science needs to be reformed.

**5.2. Clinical experiences**

Basic science courses have not been adequately integrated with clinical experiences (the mean score was 2.56 for the three universities). According to the report of Educational Development Center of Tehran University, the present curriculum lacks any integration between basic sciences (horizontal integration) and clinical science (vertical integration) (23). In Simon Watmoug et al.’s study (2009), students had complained of the gap between preclinical and clinical experiences (24). In another study by Simon Watmoug on the effectiveness of the revised curriculum entitled *Tomorrow's Doctors*, he claimed that graduates asserted that making a reform in the curriculum in the form of integration program and early clinical exposure in the very first year by making use of Clinical Skills Center made them feel far more prepared for their role as a doctor. The graduates said that making use of Clinical Skills Center was excellent and advantageous (25). In a comparative study of Iranian general practitioner training program and that of some other well-known medical colleges, Ghaffari et al. (2011) clearly said that both vertical and horizontal integration in the curriculum was made at Stanford, McGill, Indiana,  Washington, Carolina, Dundee, Oxford, Melbourne and Pretoria Universities (26). Exponential progress of science in the world and inevitable concordance of medical science with this trend has led to a need for making changes in general practitioner training program especially in basic science courses in terms of both contents and integrated layout more than before (27).

This study showed that the quality of acquired clinical experiences was at an average level. The maximum score belonged to pediatrics ward in both Shiraz and Fasa Medical Universities, but psychiatry in Jahrom Medical University. Overall, pediatric ward gained the highest score for clinical experiences in the three universities. Nevertheless, the minimum score was devoted to social medicine at the three universities. With regard to the fact that the social medicine attained the lowest score in the three universities, as it is an important course in general practitioner program, it seems essential to investigate the real cause. Khaje Azad’s study (2010) showed that the contents of the curriculum at the clinical level, based on standard national indices, were poor in the students’ opinions (21).Therefore, it is necessary that hospital wards’ training programs that contribute to clinical medical education be in the same line with the learners’ educational needs so that they can get prepared after consultation with the medical universities’ faculty members (28).

This study showed that the quality of the medical apprenticeship course was satisfactory; the departments of Anesthesiology, Psychiatry, and Surgery gained the maximum scores in Shiraz, Fasa and Jahrom Medical University, respectively. Overall, the highest score belonged to the department of Anesthesiology in the three universities. However, the department of Community Medicine had the minimum score in the three universities. The adjacency of the three universities is the point of strength by which their clinical training directors and the heads of the departments can hold sessions, exchange ideas and make use of the experiences of the departments with higher scores leading to taking effective steps in promoting clinical training level. According to the report of Educational Development Center of Tehran University, clinical training in apprenticeship and internship courses cannot provide the students with necessary self-confidence and trains them as independent general practitioners. According to this study, only 16% of the students believed that they have gained necessary self-confidence for practicing independently (23).

The knowledge tests (both written and computerized) were mostly used in the three universities. According to the Association of American Medical Colleges (22), knowledge tests are also the most widely used tests. The report of Educational Development Center of Tehran University indicates that educational assessment system more focuses on low level learning (23). As the aim of evaluation is to strengthen activities and to maximize effective methods and also to weaken or to eliminate the ineffective activities and undesirable procedures, measures should be taken to make use of modern evaluation methods due to their special structure that can act more effectively than their traditional counterparts in improving educational quality.

The researchers observed that there were students’ direct learning from clinical teachers, more communication between clinical professors and students, development of self-confidence, gaining high technical skill and independence of students in Fasa and Jahrom Medical Universities since they have no residents. In spite of the fact that Shiraz Medical University is ranked as Type One, where high school students are highly interested to study in, sometimes it is seen that when excellent students are not admitted at such universities, they would become desperate and depressed; therefore, they should be consulted about the points of strength of type two and newly established universities before choosing their majors. 

It seems that the most obvious point of weakness of Fasa and Jahrom Medical Universities which are ranked as Type Two universities is the insufficient number of experienced professors and lack of diversity of patients. The problem of such universities can be solved by increasing the number of faculty members, providing the faculty members with banking facilities, sending the students to the neighboring colleges to observe a variety of patients, employing the newly graduated doctors where they have studied, and making use of local workforce. 

**Conclusion**

Due to great advances in medical technology and paramedical sciences and the special role of doctors in international community, there is growing expectation of doctors and medical services in terms of quality and quantity. Currently, based on international standards, international community has concentrated its attention on the issue that doctors graduated today should be able to not only provide health care services but also decide wisely for patients according to different geographical, social and economic conditions. They should be skillful in establishing communication and be effective managers and efficient directors in health and community groups. They should always maintain their inward and dynamic motivation to seek knowledge and do lifetime research. It seems that general practitioner training program is facing problems and weaknesses in both pre-clinical and clinical courses. The graduates do not feel they have acquired essential readiness to enter their profession. Therefore, it seems necessary to hold an annual evaluation of the graduates in Iran and revise the Iranian general practitioner training program according to the global medical development and international standards. 
